# The Sussex Provident Scheme and Its Critics

**Published:** 1922-01

**Authors:** 


*To the Editor of* THE HOSPITAL AND HEALTH REVIEW.

### The Sussex Provident Scheme and its Critics.

*To the Editor of* THE HOSPITAL AND HEALTH REVIEW.

SIR,—In view of the fact that some doubts have been expressed as to the economic soundness and financial stability of the Sussex Provident Scheme, it may interest some of your readers to hear the results of the first eleven months' working.

It was decided to make the first financial year one of eleven months in order that the Associated Hospitals might receive what was owing to them before the last quarter day of the current year. The financial arrangement which was agreed to when the scheme was initiated was that for the first year the income of the scheme should be divided into forty parts and that these should be allotted beforehand to the Associated Hospitals, in a varying proportion, based upon the work which each might be expected to do. This was, of course, a purely experimental arrangement and subject to being changed in the light of the first year's experience, but the results which have been attained will be of value in the future whether the Scheme maintains its existence or not.

One satisfactory result is that, although some hospitals have been favoured more than others, they have all been paid in full for the cost of the services which they have rendered to the members of the Scheme during the year, but beyond this 100 per cent. the percentage of payment to the cost of the work done varies very considerably. One hospital which, so far as can be ascertained, has not been called upon for any service to the members of the Scheme has been paid one-twentieth of the total income, and of the others

